# New Short Strategy for the Synthesis of the Dibenz[*b*,*f*]oxepin Scaffold

**DOI:** 10.3390/molecules181214797

**Published:** 2013-11-29

**Authors:** David R. R. Moreno, Giorgio Giorgi, Cristian O. Salas, Ricardo A. Tapia

**Affiliations:** 1 Departamento de Química Orgánica, Facultad de Química, Pontificia Universidad Católica de Chile, Santiago 7820436, Chile; E-Mails: drmoreno@uc.cl (D.R.R.M.); cosalas@uc.cl (C.O.S.); 2 Departamento de Química Orgánica y Farmacéutica, Facultad de Farmacia, Universidad Complutense, Madrid 28040, Spain; E-Mail: giorgiogiorgi@farm.ucm.es

**Keywords:** dibenzoxepins, Wittig reaction, McMurry reaction, aromatic nucleophilic substitution

## Abstract

In this report a short and efficient synthesis of the dibenz[*b*,*f*]oxepin framework through intramolecular S_N_Ar and McMurry reactions is described. The diaryl ethers required for the McMurry reaction have been obtained in good yields under microwave-assisted conditions of the reaction of salicylaldehydes with fluorobenzaldehydes without catalysts. Application of an intramolecular McMurry reaction to the synthesized diarylethers using TiCl_4_/Zn in THF gave the target dibenzo[*b*,*f*]oxepin system in 53%–55% yields.

## 1. Introduction

**Figure 1:**
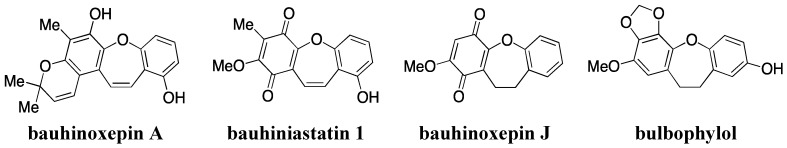
Structure of some natural dibenz[*b*,*f*]oxepins.

The dibenz[*b*,*f*]oxepin scaffold is an important synthetic target because a large number of compounds having this skeleton present relevant biological activities; such as antidepressant [1], anxiolytic [2], antipsychotic [3,4], angiotensin-II-receptor-antagonist [5], and anti-inflammatory properties [6]. Additionally, a number of natural occurring dibenz[*b*,*f*]oxepins have been isolated from plants of the genus *Bauhinia* (fam. Fabaceae) and many of them also exhibit important biological activities [7]. For example, bauhinoxepin A isolated from *Bauhinia saccocalyx* Pierre (Figure 1), shows antimycobacterial activity [8]. Pettit *et al*. isolated bauhiniastatin 1 from *Bauhinia purpurea* L., which exhibits significant growth inhibition activity against several human cancer lines [9]. From the same plant, Kittakoop *et al*. have described bauhinoxepin J, which shows potent antimycobacterial and antimalarial activities, as well as tumor growth inhibitory activity, against KB cells [10]. Bulbophylol B is another interesting example isolated from *Bulbophyllum kwangtungense* Schlecht (fam. Orchidaceae), which displays significant cytotoxicity against human epithelial carcinoma (HeLa) and human erythromyeloblastoid leukemia (K562) cell lines [11].

Synthetic approaches to natural dibenzo[*b*,*f*]oxepins have been directed mainly to the preparation of dihydro derivatives. For example, the total synthesis of bauhinoxepin J using an intramolecular persulfate-mediated radical addition to a quinone was described by Krauss and Kim [12]. Furthermore, Katoh *et al*. [13] have also recently described their synthesis using the reaction of an aryllithium derivative with a phenylacetaldehyde and subsequent internal nucleophilic addition/elimination sequences as key steps. Yao *et al*. have described the synthesis of bulbophylol employing Wittig, selective reduction and intramolecular Ullmann reactions as key steps (18% overall yield over 12 steps) [14]. It is noteworthy that the synthesis of bauhinoxepin A and bauhiniastatin 1 are not reported, probably because the described routes to dibenzo[*b*,*f*]oxepins are multi-step procedures or require the preparation of complex starting materials [15]. Some interesting approaches have been described recently, but they are limited to the synthesis of dibenz[*b*,*f*]oxepincarboxylic acid derivatives [16,17]. In connection with our interest on the synthesis of bioactive heterocyclic quinones [18,19], herein we describe a convenient procedure for the preparation of the dibenzo[*b*,*f*]oxepin scaffold.

**Scheme 1:**
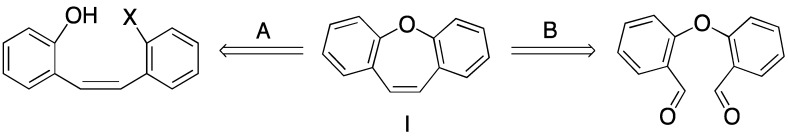
Strategies for the synthesis of dibenzo[*b*,*f*]oxepin scaffold.

Retrosynthetic analysis of the tricyclic system **I** led us to consider two strategies (Scheme 1). Approach A, is via an intramolecular Ullmann, or nucleophilic aromatic substitution (S_N_Ar) reaction [20]. Path B, was envisaged through an intramolecular McMurry reaction, which has been used successfully in the synthesis of natural products [21], but there are no precedents for the preparation of dibenzo[*b*,*f*]oxepins using it, except for sulfur and selenium analogues [22].

## 2. Results and Discussion

**Scheme 2:**
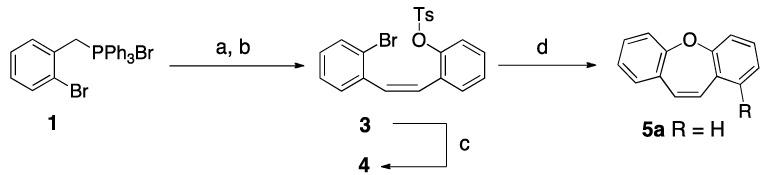
Synthesis of dibenzoxepin **5a** by intramolecular S_N_Ar reaction.

First, we focused our research on the synthesis of a *Z*-stilbene. To achieve our objective, we planned to apply the Wittig reaction that gives high *Z* selectivity when both the ylide and benzaldehyde incorporate *ortho*-halo and *ortho*-alkoxy substituents [23,24]. Thus, Wittig reaction of *o*-bromobenzyl-triphenylphosphonium salt **1** [23] with 2-formylphenyl-4-methylbenzene sulfonate (**2**) in the presence of potassium *t*-butoxide gave stilbene **3** (87%) as a single isomer. Attempts to obtain dibenzo[*b*,*f*]oxepin **5a** directly from compound **3**, by applying an intramolecular palladium-catalyzed biaryl ether formation using Pd(OAc)_2_ and tri(*o*-tolyl)phosphine as described by Harayama *et al*. for an aza-analog [25] were unsuccessful. Cleavage of the *p*-toluenesulfonate group under standard basic conditions (KOH/EtOH-H_2_O) gave phenol **4** which was directly converted to dibenzo[*b*,*f*]oxepin **5a** (72%) by treatment with cesium carbonate in DMSO at 180 °C under microwave irradiation (Scheme 2).

*Reagents and conditions*: (a) *t*-BuOK, THF, 0 °C, 30 min; (b) **2**, THF, 18 h, rt; (c) KOH, EtOH-H_2_O, 1 h reflux; (d) DMSO, Cs_2_CO_3_, MW, 180 °C,15 min.

**Scheme 3:**
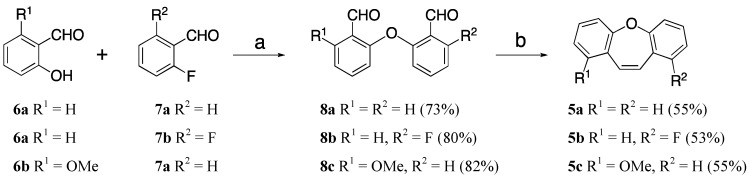
Synthesis of dibenzo[*b*,*f*]oxepin scaffold via McMurry reaction.

Considering the low synthetic efficiency of the Wittig process, we focused our attention on the intramolecular McMurry reaction. Therefore, we concentrated our attention on the preparation of suitable diaryl ether precursors. The synthesis of *o*-phenoxybenzaldehydes by Ullmann or S_N_Ar nucleophilic aromatic substitution reactions of salicylaldehydes with aryl halides normally requires harsh conditions, long reaction times and often gives low yields [26,27,28]. Considering the successful application of microwave irradiation to improve the nucleophilic S_N_Ar reaction of activated aryl halides with phenols [29], we decided to use this methodology to obtain diaryl ethers **8**. Therefore, preliminary experiments were carried out in order to determine the optimal conditions for the synthesis of **8a** using highly polar solvents such as DMSO or DMA and K_2_CO_3_ or Cs_2_CO_3_ as bases [20]. The reaction of 1.2 equivalents of 2-hydroxybenzaldehyde (**6a**) with 1.0 equivalent of 2-fluoro-benzaldehyde (**7a**) and K_2_CO_3_ (2 equiv.) in DMSO using microwave irradiation over a wide temperature range was examined. The best result was obtained when the reaction was carried out at 120 °C (Table 1, entry 3) with a 73% yield of dialdehyde **8a** and at higher temperatures a progressive degradation of compound **8a** was observed. Similar results were obtained using DMA and Cs_2_CO_3_ as solvent and base, respectively (Table 1, entry 14). Using the optimized conditions, 2-fluoro-6-(2-formylphenoxy)-benzaldehyde (**8b**) and 2-(2-formylphenoxy)-6-methoxybenzaldehyde (**8c**) were obtained in 80% and 82% yield. Finally, the treatment of dialdehyde **8a** with TiCl_4_ (3.0 equiv.) and Zn (6.0 equiv.) in THF at reflux for 2.5 h gave compound **5a** in 55% yield through an intramolecular McMurry coupling reaction. Similarly, dialdehydes **8b** and **8c** underwent intramolecular McMurry coupling to give dibenzoxepins **5b** and **5c** (53%–55%) (Scheme 3).

**Table 1:** Optimization of microwave-induced synthesis of 2,2'-oxybis(benzaldehyde)**8a**.

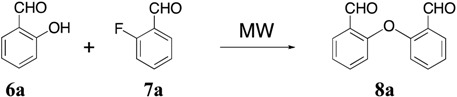					
Entry	Temp. (°C)	Base	Solvent	Time (min)	Yields (%)
1	100 °C	K_2_CO_3_	DMSO	30	48%
2	110 °C	K_2_CO_3_	DMSO	30	67%
3	120 °C	K_2_CO_3_	DMSO	30	73%
4	130 °C	K_2_CO_3_	DMSO	30	72%
5	140 °C	K_2_CO_3_	DMSO	30	55%
6	160 °C	K_2_CO_3_	DMSO	30	3% ^a^
7	100 °C	Cs_2_CO_3_	DMSO	30	50%
8	110 °C	Cs_2_CO_3_	DMSO	30	65%
9	120 °C	Cs_2_CO_3_	DMSO	30	73%
10	130 °C	Cs_2_CO_3_	DMSO	30	70%
11	140 °C	Cs_2_CO_3_	DMSO	30	45%
12	160 °C	K_2_CO_3_	DMA	30	0% ^a^
13	120 °C	K_2_CO_3_	DMA	30	72%
14	120 °C	Cs_2_CO_3_	DMA	30	73%
15	120 °C	K_2_CO_3_	DMA	24 h	71% ^b^

^a^ The decomposition of the product was observed; ^b^ Reaction performed without microwave irradiation.

*Reagents and conditions*: (a) DMSO, MW, 120 °C 30 min; (b) Zn, TiCl_4_, THF, 12 h rt.

**Scheme 4:**
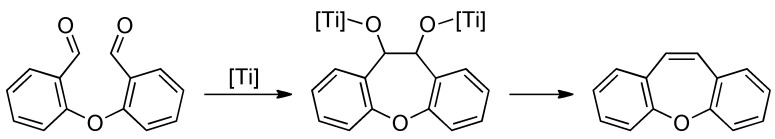
Possible mechanism for the McMurry reaction formation of dibenzo[*b*,*f*]oxepins.

The mechanism of the McMurry reaction is still under debate, but new evidences suggest participation of a metallopinacol intermediate formed by dimerization of ketyl radicals [30,31,32,33]. A possible mechanism for our route is shown in Scheme 4.

## 3. Experimental

### 3.1. General

Melting points were measured on a Stuart Scientific SMP3 apparatus (Stuart Scientific, Manchester, UK) and are uncorrected. Infrared (IR) spectra (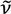
_max_) were recorded on a Bruker Model Vector 22 spectrophotometer (Bruker Optik GmbH, Bremen, Germany). ^1^H- (400 MHz) and ^13^C-NMR (100 MHz) spectra were obtained on a Bruker AM-400 instrument (Bruker BioSpin GmbH, Rheinstetten, Germany), using tetramethylsilane as internal reference. Column chromatography was performed on silica gel Merck 60 (70–230 mesh) (Merck, Darmstadt, Germany). High-resolution mass spectrum was obtained using a Thermo Finnigan mass spectrometer Model MAT 95XP (Thermo Finnigan, San Jose, CA, USA). Microwave-assisted reactions were carried out in an Anton Paar Monowave 300 Microwave Synthesis Reactor (Anton Paar GmbH, Graz, Austria) in 30 mL sealed vials. THF was freshly distilled over sodium. DMSO and DMA were dried over 4 Å molecular sieves prior to use. Cs_2_CO_3_ and K_2_CO_3_ were dried overnight at 200 °C prior to use. All other reagents were used without further purification.

*(Z)-2-(2-Bromostyryl)phenyl 4-methylbenzenesulfonate* (**3**). *t*-BuOK (157 mg, 1.4 mmol) was added to a suspension of phosphonium salt **1** (615 mg, 1.2 mmol) in THF (15 mL), at 0 °C and under a nitrogen atmosphere. The mixture was stirred at 0 °C for 30 min and a solution of aldehyde **2** (276.3 mg, 1.0 mmol) in THF (10 mL) was added via syringe. The reaction mixture was allowed to warm to room temperature and stirred for 18 h. The cooled reaction mixture was poured into water (30 mL) and extracted with EtOAc (3 × 20 mL). The combined organic layers were washed with brine, dried with MgSO_4_ and concentrated under reduced pressure. The crude product was purified by silica gel flash chromatography (CH_2_Cl_2_-hexane, 1:9) to give stilbene **3** (375 mg, 87%) as colorless oil. IR (KBr): 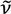
_max_ 3061, 1597, 1444, 1366, 1262, 1025, 810, 722, 671 cm^−1^. ^1^H-NMR (CDCl_3_): δ 2.39 (s, 3H), 6.56 (d, *J* = 12 Hz, 1H), 6.61 (d, *J* = 12 Hz, 1H), 6.75 (d, *J* = 8 Hz, 1H), 6.90–7.10 (m, 6H), 7.29 (d, *J* = 8 Hz, 2H), 7.54 (d, *J* = 8 Hz, 1H), 7.80 (d, *J* = 8 Hz, 2H). ^13^C-NMR (acetone-*d_6_*): δ 22.1, 124.0, 124.8, 126.7, 128.0, 128.4, 130.0 (2C), 130.3, 130.6, 131.4 (2C), 131.7, 131.9, 132.1, 132.6, 133.9, 134.2, 138.3, 147.3, 149.0. HRMS (EI): *m*/*z* [M^+^] calcd for C_21_H_17_BrO_3_S: 428.0082; found: 428.0077.

*Dibenz[b,f]oxepin* (**5a**). Stilbene **3** (215 mg, 0.5 mmol) was added to a solution of KOH (900 mg, 16 mmol) in a mixture of EtOH (15 mL) and H_2_O (15 mL) and the suspension was heated under reflux for 1 h. After cooling, the reaction mixture was acidified with aqueous HCl (10%) to pH 4 and extracted with CH_2_Cl_2_ (3 × 25 mL). The combined organic extracts were washed with saturated aqueous NaHCO_3_, dried, and filtered through a short column of silica gel. After the removal of the solvent, the residue was dissolved in DMSO (5.0 mL) and Cs_2_CO_3_ (651.6 mg, 2.0 mmol) was added. The reaction mixture was heated in a microwave reactor at 180 °C for 15 min. After cooling, the solvent was evaporated under reduced pressure and the crude product was purified by flash column chromatography (silica gel, EtOAc-hexanes; 1:9) to afford **5a** (70 mg, 72%), mp 108.5–109.5 °C (Lit. 106–108 °C [34], 110–111 °C [35]). IR (KBr): 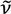
_max_ 3069, 3044, 1483, 798 cm^−1^. ^1^H-NMR (acetone-*d_6_*) δ 6.82 (s, 2H), 7,19 (t, *J* = 7,8 Hz, 2H), 7.25 (d, *J* = 7.8 Hz, 2H), 7.30 (d, *J* = 7.8 Hz, 2H), 7.38 (t, *J* = 7.8 Hz, 2H). ^13^C-NMR (acetone-*d_6_*) δ 122.9, 126.6, 131.1, 131.6, 131.7, 132.3, 159.1.

### 3.2. General Procedure for the Preparation of Diarylethers **8**

A mixture of hydroxybenzaldehyde **6a** (1.2 mmol), fluorobenzaldehyde **7** (1.0 mmol), cesium carbonate (1.30 g, 4.0 mmol) and DMSO (4.0 mL) in a 30 mL microwave vial was irradiated at 120 °C for 30 min under nitrogen. The reaction mixture was diluted with dichloromethane (20 mL), washed with brine (3 × 10 mL), dried (MgSO_4_) and evaporated. The residue was purified by flash column chromatography (EtOAc-hexanes, 1:9).

*2,2'-Oxybis(benzaldehyde)* (**8a**). Following general procedure, from 2-hydroxybenzaldehyde (146.5 mg, 1.2 mmol) and 2-fluorobenzaldehyde (124.1 mg, 1.0 mmol) compound **8a** was obtained (168 mg, 74%), mp 76–77 °C (Lit. 74 °C [36], 77.0–77.5 °C [37]). IR (KBr): 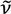
_max_ 1686, 1574, 1473, 1454, 1393, 1301, 1224, 760 cm^−1^. ^1^H-NMR (acetone-*d_6_*): δ 7.11 (d, *J* = 8.3 Hz, 2H), 7.39 (t, *J* = 7.7 Hz, 2H), 7.72 (m, 2H), 7.96 (dd, *J* = 7.7, 1.5 Hz, 2H), 10.53 (s, 2H), ^13^C-NMR (acetone-*d_6_*): δ 121.6, 126.7, 129.6, 131.0, 138.4, 161.2, 190.9.

*2-Fluoro-6-(2-formylphenoxy)benzaldehyde* (**8b**). Following the general procedure, from 2-hydroxybenzaldehyde (146.5 mg, 1.2 mmol) and 2,6-difluorobenzaldehyde (142.1 mg, 1.0 mmol) compound **8b** was obtained (196 mg, 80%), mp 80–81 °C. IR (KBr): 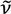
_max_ 1685, 1611, 1598, 1577, 1396, 787 cm^−1^. ^1^H-NMR (CDCl_3_) δ 6.73 (d, *J* = 8.3 Hz, 1H), 6.99 (m, 2H), 7.33 (t, *J* = 7.5 Hz, 1H), 7.54 (m, 1H), 7.62 (ddd, *J* = 8.3, 7.5, 1.5 Hz, 1H), 7.99 (dd, *J* = 7.5, 1.5 Hz, 1H), 10.44 (s, 1H), 10.51 (s, 1H), 10.43 (s, 1H). ^13^C-NMR (CDCl_3_) δ 112.5, 112.7, 115.0, 115.1, 119.7, 125.3, 127.7, 129.8, 136.4, 136.5, 158.5, 159.0, 186.2, 188.9. HRMS (EI): *m*/*z* [M^+^] calcd for C_14_H_9_FO_3_: 244.0536; found: 244.0532.

*2-Hydroxy-6-methoxybenzaldehyde* (**6b**). A solution of 2,6-dimethoxybenzaldehyde (2.0 g, 12 mmol) in CH_2_Cl_2_ (20 mL) was added dropwise to a stirred suspension of AlCl_3_ (2.4 g, 18 mmol) in CH_2_Cl_2_ (30 mL) at −20 °C. The reaction mixture was allowed to warm to room temperature and then stirred for 6 h. After the addition of 6 M HCl (20 mL) the biphasic mixture was stirred vigorously for 12 h and the aqueous solution was extracted with CH_2_Cl_2_ (3 × 20 mL) The combined organic extracts were washed with water, brine, dried (MgSO_4_) and evaporated under reduced pressure. Purification of the residue by flash chromatography on silica gel (EtOAc-hexanes; 1:9) gave compound **6b** (1.7 g, 93%), mp 72–74 °C (Lit. 73–75 °C [38]).

*2-(2-Formylphenoxy)-6-methoxybenzaldehyde* (**8c**). Following general procedure, from 2-hydroxy-6-methoxybenzaldehyde (182.6 mg, 1.2 mmol) and 2-fluorobenzaldehyde (124.1 mg, 1.0 mmol) compound **8a** was obtained (210 mg, 82%), mp 116–117 °C. IR (KBr): 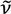
_max_ 2914, 2862, 2762, 1687, 1600, 1279, 1236, 758, 737 cm^−1^. ^1^H-NMR (CDCl_3_) δ 3.97 (s, 3H), 6.54 (d, *J* = 8.4 Hz, 1H), 6.83 (d, *J* = 8.4 Hz, 1H), 6.86 (dd, *J* = 8.4, 1.8 Hz, 1H), 7.22 (tt, *J* = 7.8, 1.8 Hz, 1H), 7.48 (t, *J* = 8.4 Hz, 1H), 7,52 (ddd, *J* = 8.4, 7.8, 1.8 Hz, 1H), 7.95 (dd, *J* = 7.8, 1.8 Hz, 1H), 10.48 (s, 1H), 10.52 (s, 1H). ^13^C-NMR (CDCl_3_) δ 56.7, 108.0, 112.5, 117.4, 118.8, 124.3, 127.4, 129.1, 136.1, 136.2, 158.3, 159.7, 163.1, 188.4, 189.5. HRMS (EI): *m*/*z* [M^+^] calcd for C_15_H_12_O_4_: 256.0736; found: 256.0733.

### 3.3. General Procedure for the McMurry Reaction

To a stirred suspension of zinc powder (98.1 mg, 3.0 mmol) in anhydrous THF (40 mL) cooled to −5 °C under an argon atmosphere, TiCl_4_ (284.5 mg, 1.5 mmol) was slowly added via syringe keeping the temperature under 0 °C. The reaction mixture was allowed to warm to room temperature and then heated at reflux for 2.5 h. The reaction was quenched with saturated NH_4_Cl solution and extracted with CH_2_Cl_2_ (3 × 20 mL). The combined organic layers were dried (MgSO_4_) and concentrated. The crude material was purified by flash chromatography (hexanes) to give the desired product.

*Dibenzo[b,f]oxepin* (**5a**). Following the general procedure, from *2,2'-oxybis(benzaldehyde)* (**8a**) (226.2 mg, 1.0 mmol) compound **5a** was obtained (107 mg, 55%), mp 108.5–109.5 °C.

*1-Fluorodibenzo[b,f]oxepin* (**5b**). Following general procedure, from 2-fluoro-6-(2-formylphenoxy)-benzaldehyde (**8b**) (244.2 mg, 1.0 mmol) compound **5a** was obtained (115 mg, 54%), mp 40.5–41.5 °C. IR (KBr): 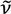
_max_ 3051, 1613, 1571, 1442, 1259, 1007, 774 cm^−1^. ^1^H-NMR (acetone-*d_6_*) δ 6.93 (d, *J* = 11.5 Hz, 1H), 6.97 (d, *J* = 11.5 Hz, 1H), 7.03 (ddd, *J* = 9.6, 8.4, 1.2 Hz, 1H), 7.13 (dt, *J* = 8.4, 1.2 Hz, 1H), 7.23 (dd, *J* = 7.6, 1.2 Hz, 1H), 7.35 (dd, *J* = 7.6, 1.7 Hz, 1H). ^13^C-NMR (acetone-*d_6_*) δ 112.3, 117.4, 120.0, 121.8, 122.6, 126.6, 129.9, 130.4, 130.5, 130.9, 131.4, 157.7, 159.5, 161.5. HRMS (EI): *m*/*z* [M^+^] calcd for C_14_H_9_FO: 212.0637; found: 212.0640.

*1-methoxydibenzo[b,f]oxepin* (**5c**). Following general procedure, from 2-(2-formylphenoxy)-6-methoxybenzaldehyde (**8c**) (256.3 mg, 1.0 mmol) compound **5c** was obtained (124 mg, 55%), mp 63–64 °C. IR (KBr): 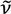
_max_ 1599, 1570, 1464, 1075, 778 cm^−1^. ^1^H-NMR (acetone-*d_6_*) δ 3.90 (s, 3H), 6.84 (d, *J* = 11.6 Hz, 1H), 6.89 (d, *J* = 8.3 Hz, 1H), 7.06 (d, *J* = 11.6 Hz, 1H), 7.20 (td, *J* = 7.4, 1.6 Hz, 1H), 7.24 (m, 1H), 7.30 (dd, *J* = 7.6, 1.6 Hz, 2H), 7.35 (t, *J* = 8.4 Hz, 1H), 7.38 (ddd, *J* = 8.4, 7.4, 1.6 Hz, 1H). ^13^C-NMR (CDCl_3_) δ 56.2, 107.3, 114.1, 120.1, 121.6, 125.1, 125.2, 129.4, 129.5, 129.9, 130.3, 131.5, 157.5, 157.8, 159.53. HRMS (EI): *m*/*z* [M^+^] calcd for C_15_H_12_O_2_: 224.0837; found: 224.0831.

## 4. Conclusions

In conclusion, we have developed a short synthesis of dibenzo[*b*,*f*]oxepin derivatives using S_N_Ar and intramolecular McMurry reactions. An efficient process to obtain diarylethers through S_N_Ar reaction of salicylaldehydes with fluorobenzaldehydes using microwave irradiation is described. McMurry reaction of diarylethers using TiCl_4_ and Zn in THF afforded the target tricyclic system in reasonable yields (53%–55%). Further work on the synthesis of natural and pharmacologically active dibenzo[*b*,*f*]oxepins are under way.

## Supplementary Materials

Supplementary materials can be accessed at: http://www.mdpi.com/1420-3049/18/12/14797/s1.
