# Supplementary material for: New Short Strategy for the Synthesis of the Dibenz[b,f]oxepin Scaffold
Source: Molecules. 2013 Nov 29;18(12):14797–806. doi: 10.3390/molecules181214797 (PMC6270238; doi:10.3390/molecules181214797)

Supplementary Information

Figure S1. ^{1}H-NMR of (Z)-2-(2-bromostyryl)phenyl 4-methylbenzenesulfonate (**3**).

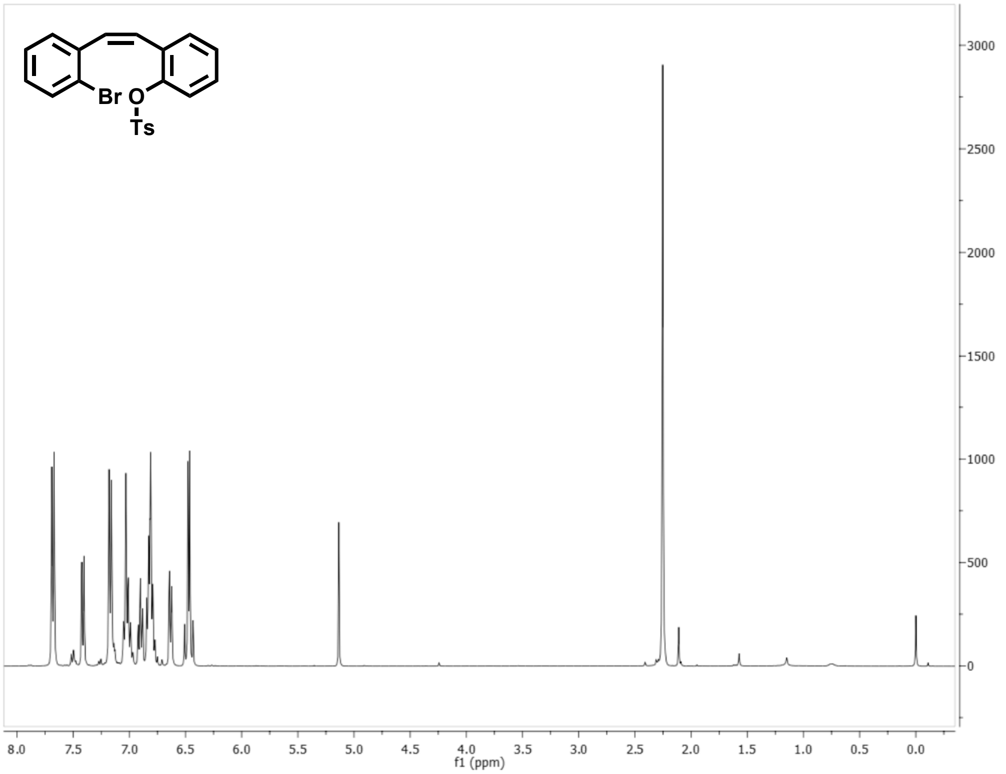

Figure S2. ^{13}C-NMR of (Z)-2-(2-bromostyryl)phenyl 4-methylbenzenesulfonate (**3**).

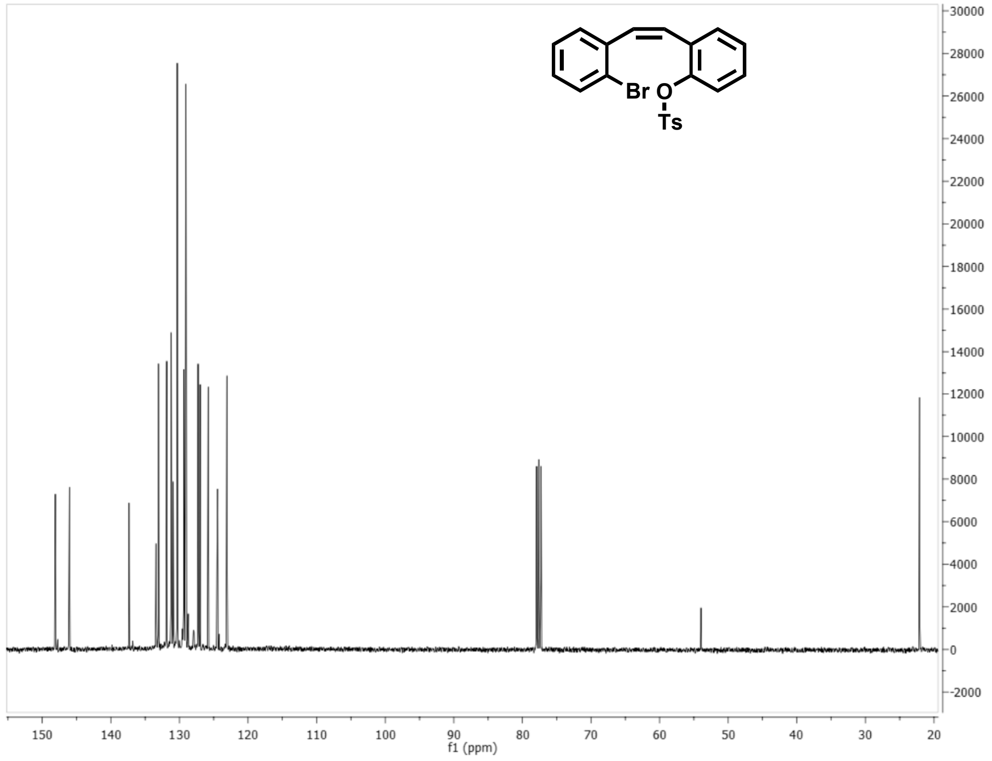

Figure S3. ^{1}H-NMR of Dibenz[b,f]oxepin (**5a**).

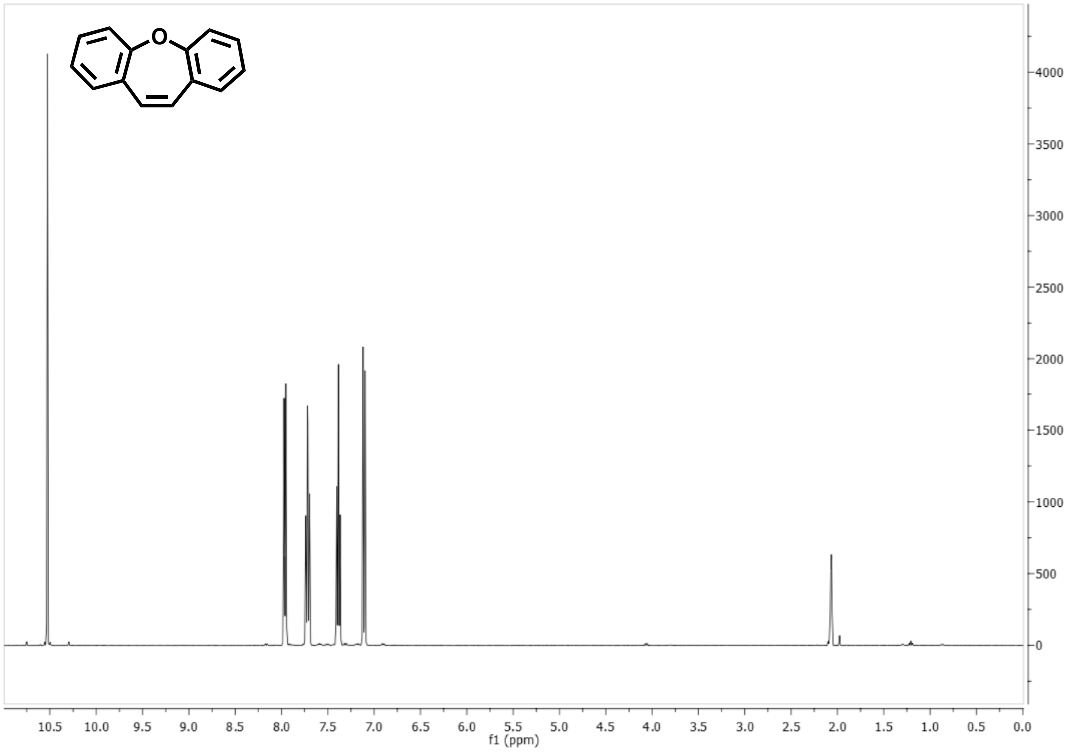

Figure S4. ^{13}C-NMR of Dibenz[b,f]oxepin (**5a**).

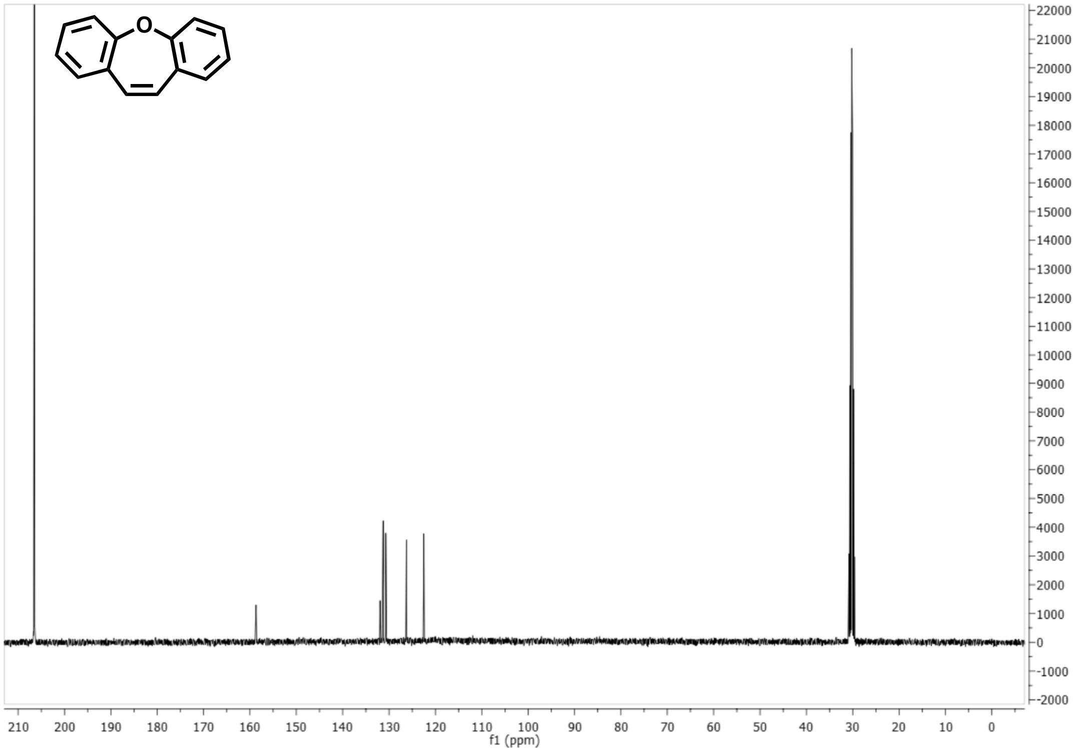

Figure S5. ^{1}H-NMR of 2,2'-oxybis(benzaldehyde) (**8a**).

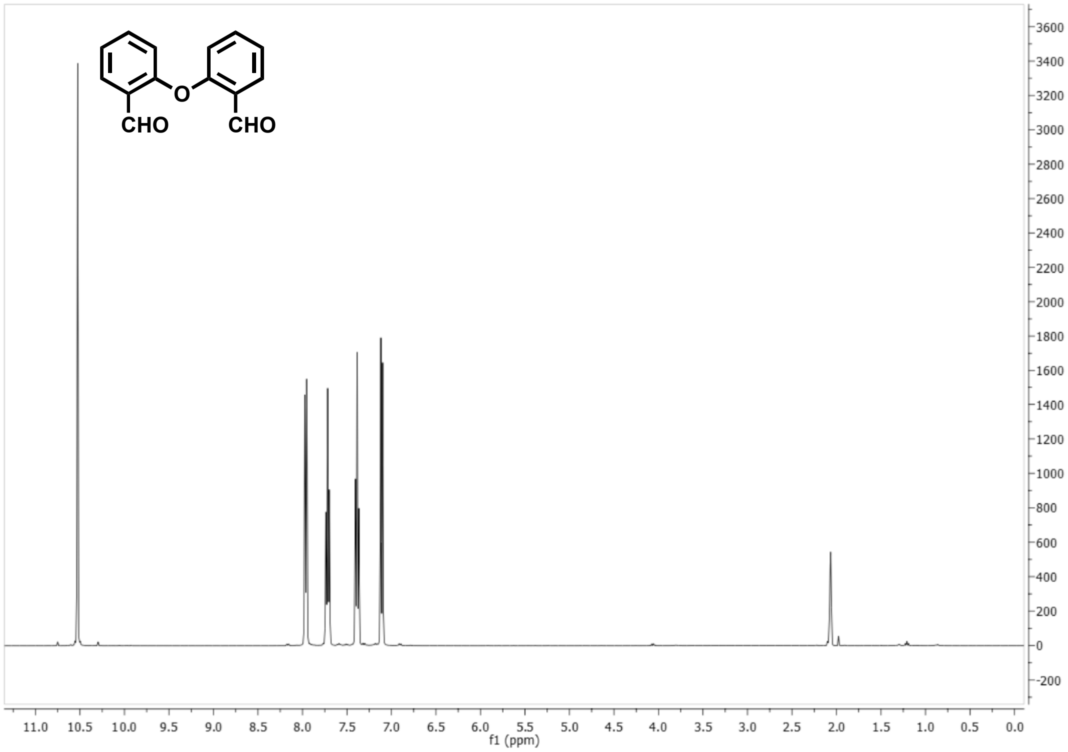

Figure S6. ^{13}C-NMR of 2,2'-oxybis(benzaldehyde) (**8a**).

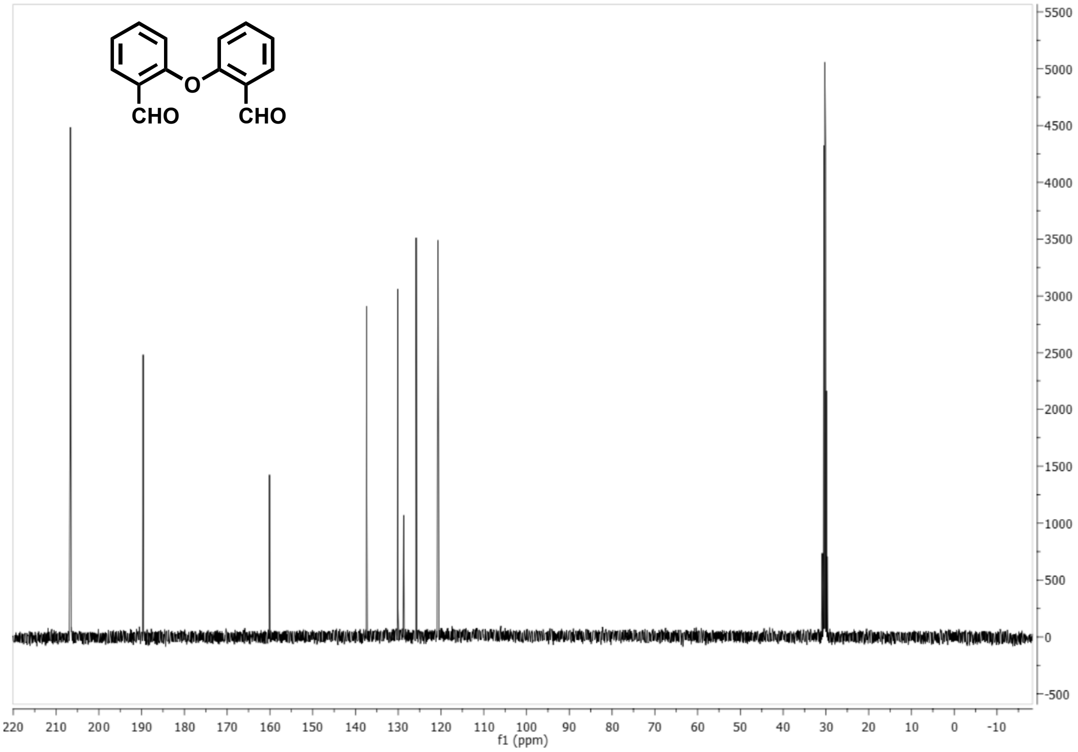

Figure S7. ^{1}H-NMR of 2-fluoro-6-(2-formylphenoxy)benzaldehyde (**8b**).

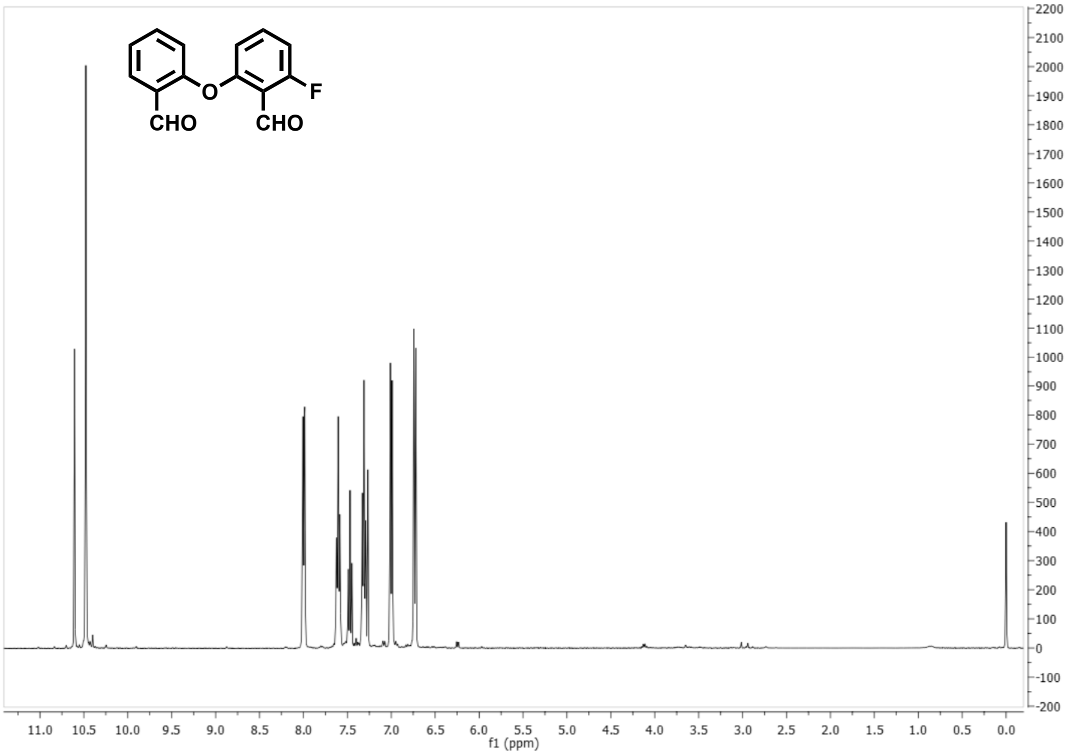

Figure S8. ^{13}C-NMR of 2-fluoro-6-(2-formylphenoxy)benzaldehyde (**8b**).

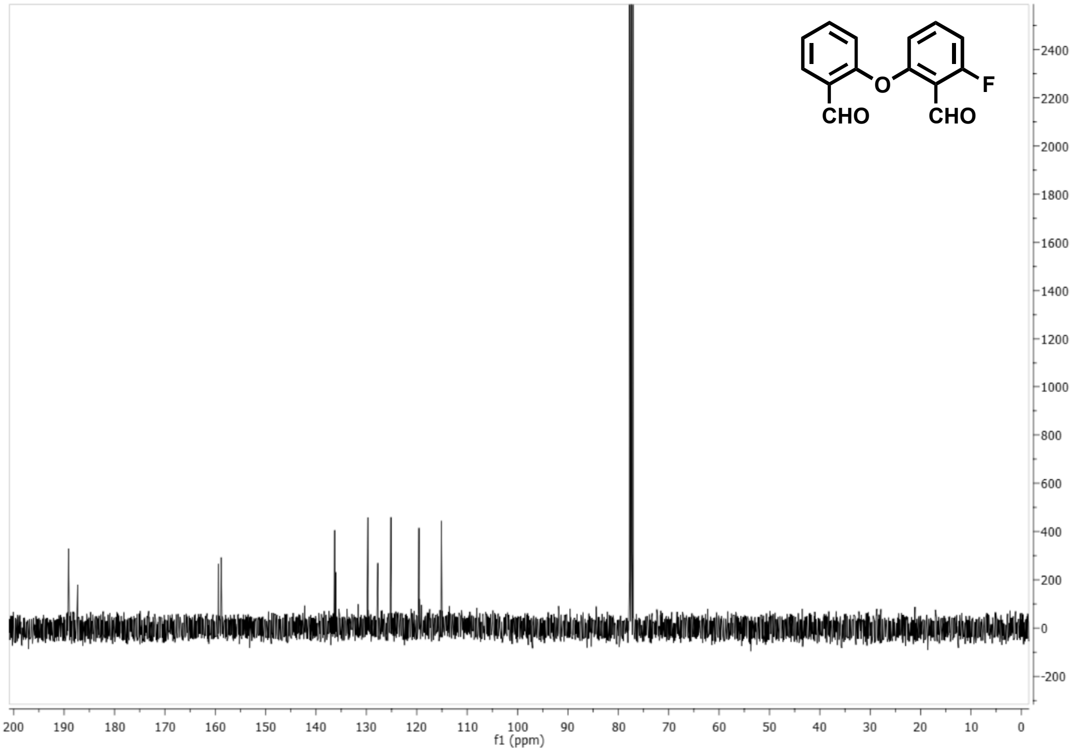

Figure S9. ^{1}H-NMR of 2-hydroxy-6-methoxybenzaldehyde (**6**).

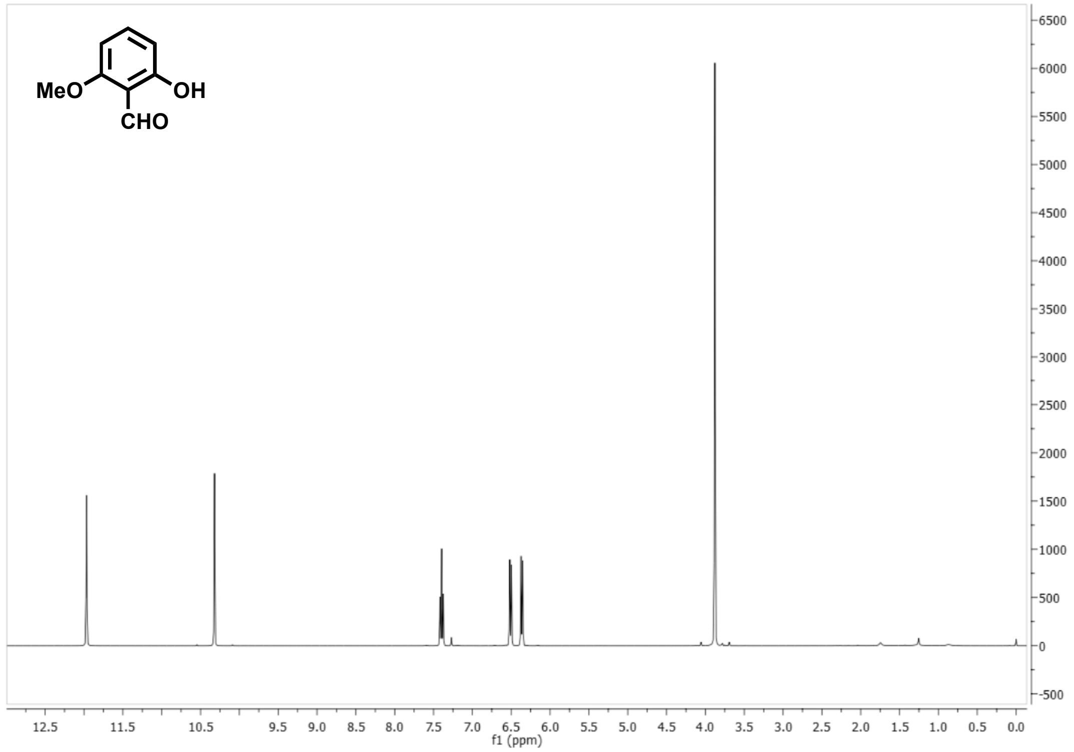

Figure S10. ^{13}C-NMR of 2-hydroxy-6-methoxybenzaldehyde (**6**).

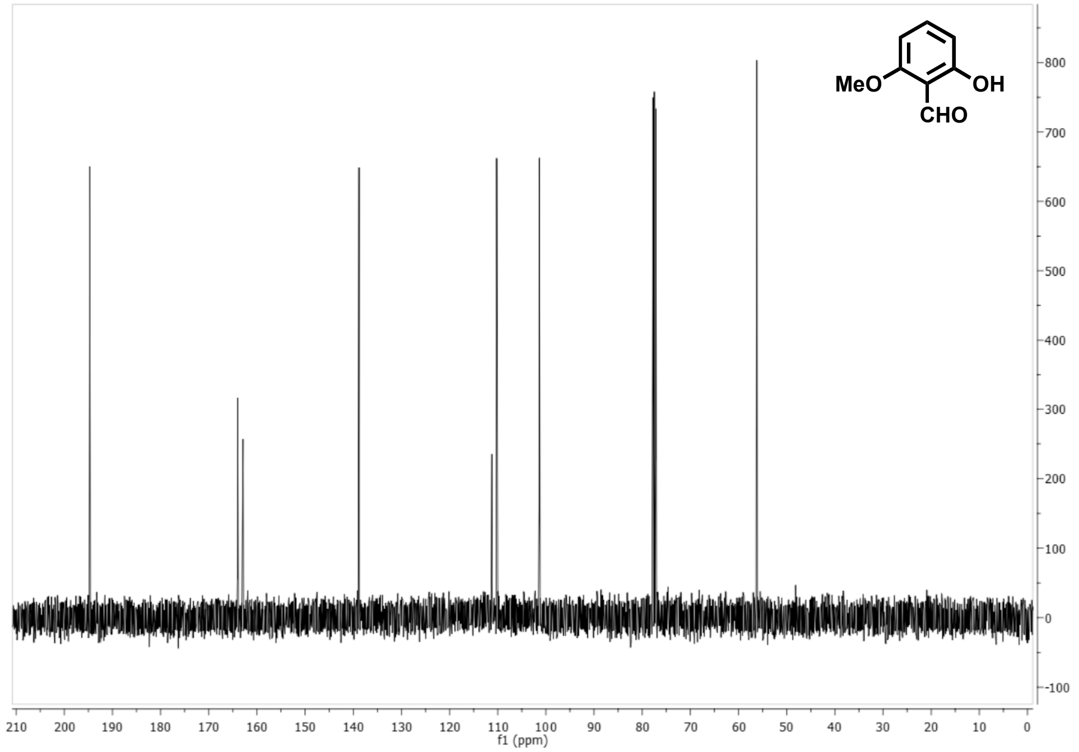

Figure S11. ^{1}H-NMR of 2-(2-formylphenoxy)-6-methoxybenzaldehyde (**8c**).

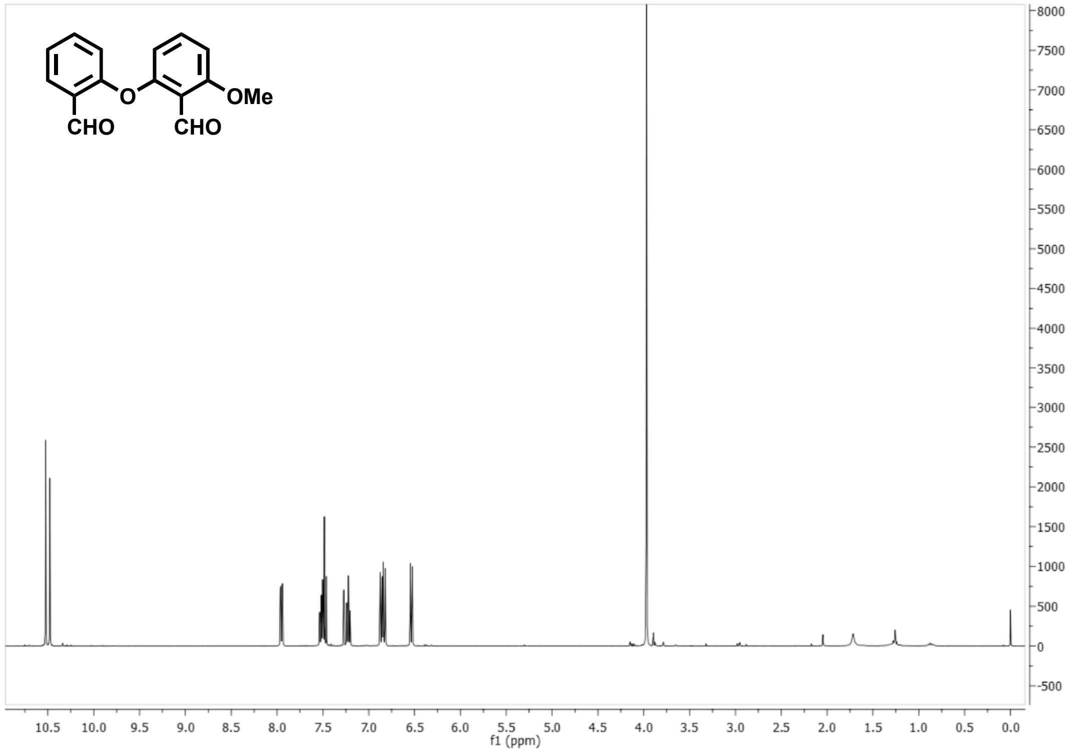

Figure S12. ^{13}C-NMR of 2-(2-formylphenoxy)-6-methoxybenzaldehyde (**8c**).

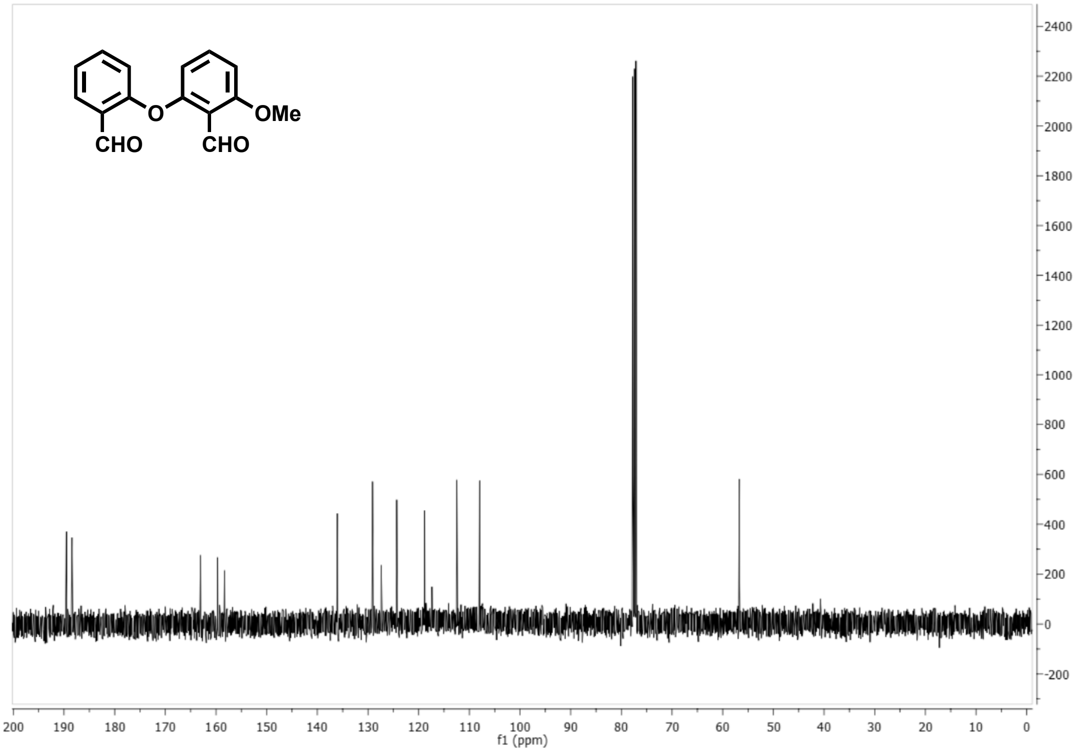

Figure S13. ^{1}H-NMR of 1-fluorodibenzo[b,f]oxepin (**5b**).

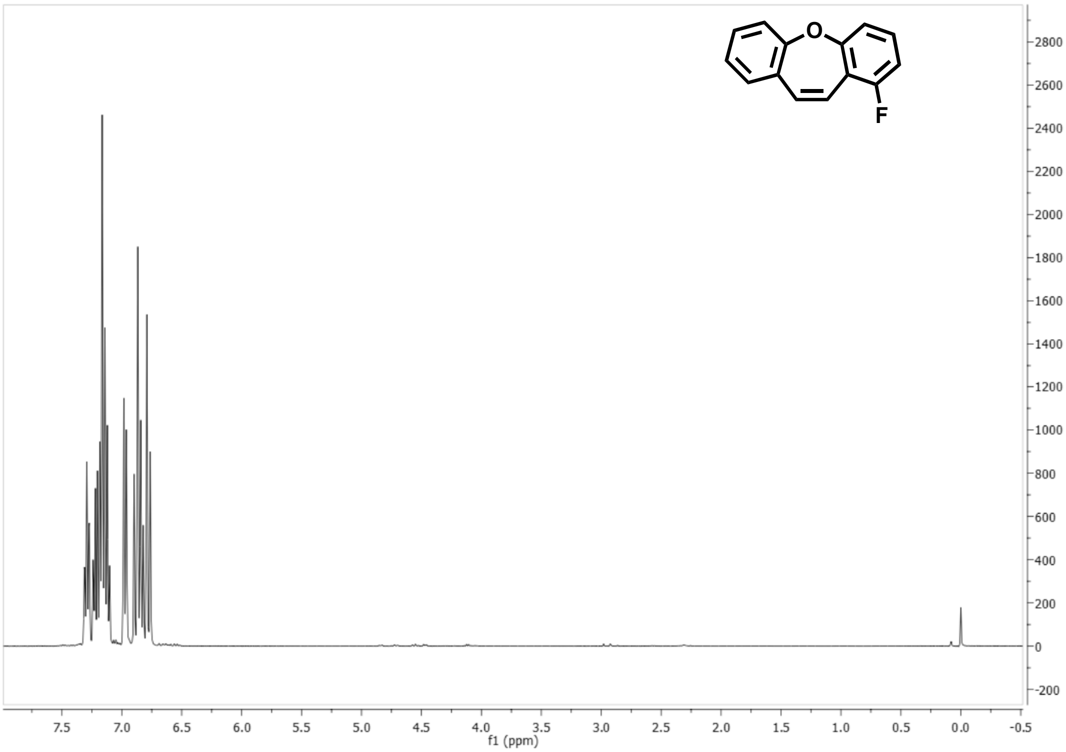

Figure S14. ^{13}C-NMR of 1-fluorodibenzo[b,f]oxepin (**5b**).

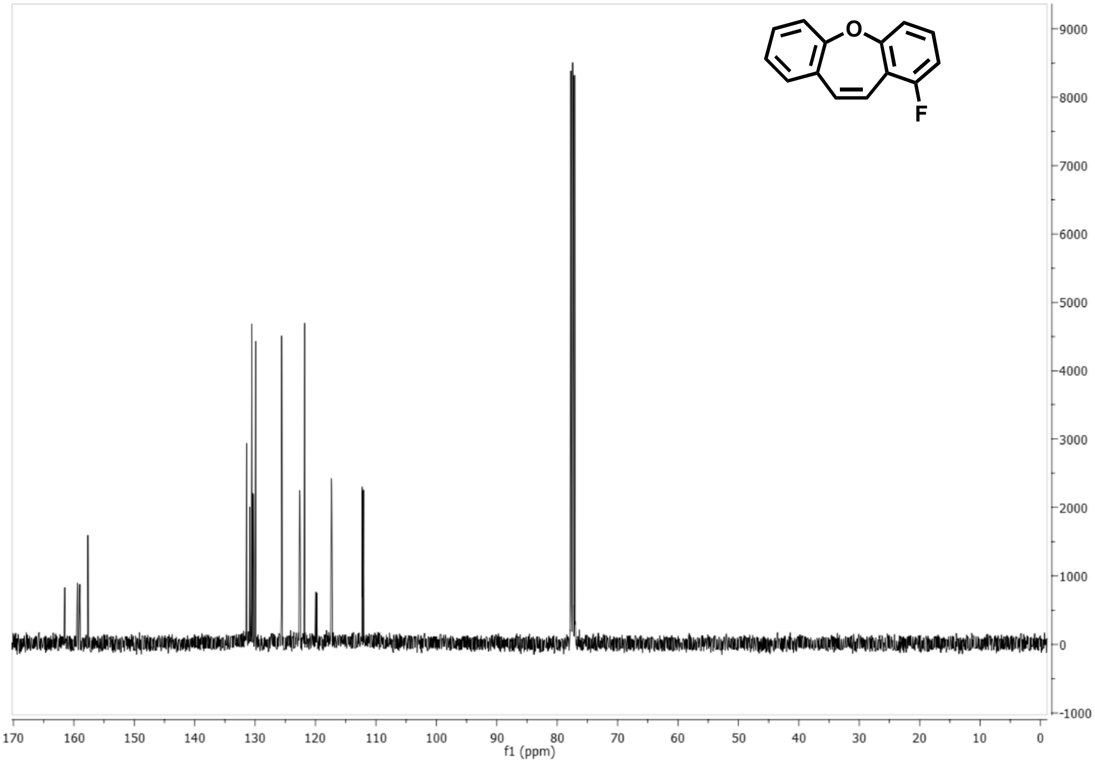

Figure S15. ^{1}H-NMR of 1-methoxydibenzo[b,f]oxepin (**5c**).

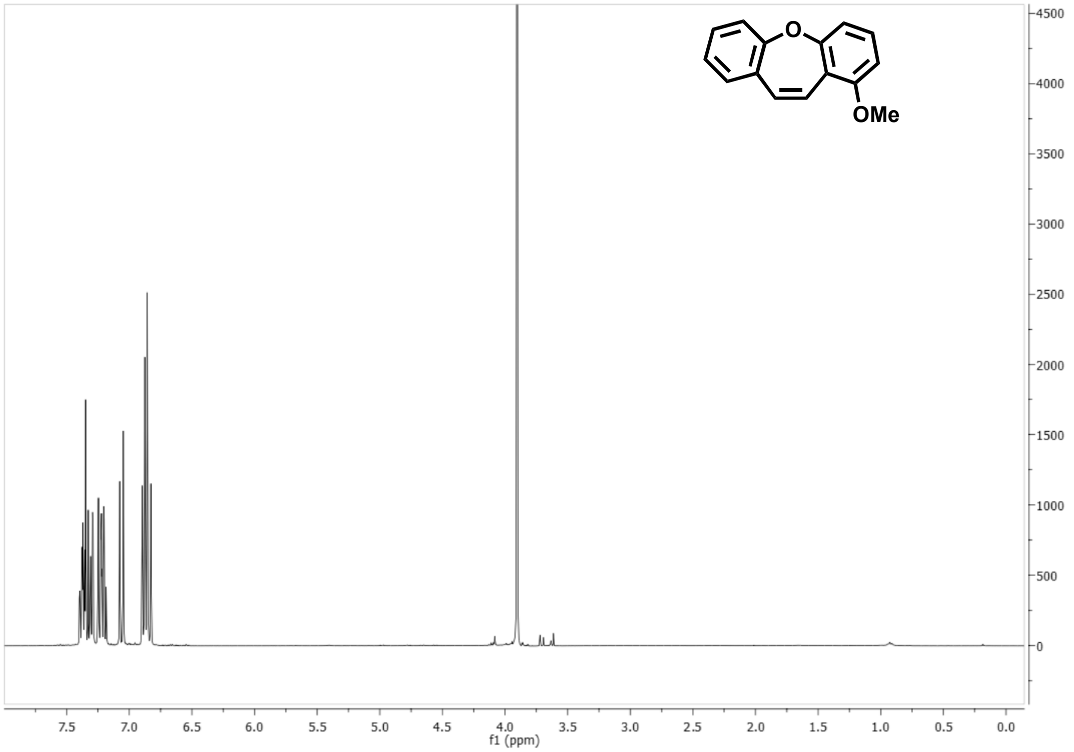

Figure S16. ^{13}C-NMR of 1-methoxydibenzo[b,f]oxepin (**5c**).

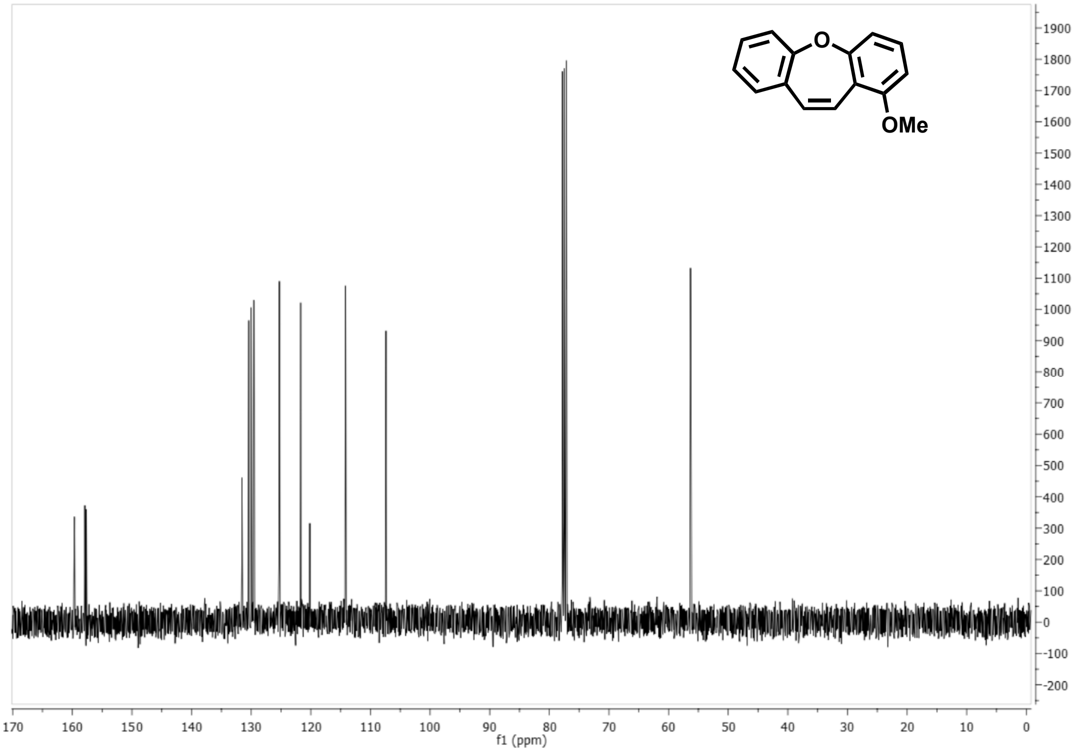

Supplement: Supplementary file 1 [file molecules-18-14797-s001.pdf]
